# Longitudinal Evaluation of Aortic Hemodynamics in Marfan Syndrome: New Insights from a 4D Flow Cardiovascular Magnetic Resonance Multi-Year Follow-Up Study

**DOI:** 10.1186/s12968-017-0347-5

**Published:** 2017-03-22

**Authors:** Julia Geiger, Daniel Hirtler, Kristina Gottfried, Ozair Rahman, Emilie Bollache, Alex J. Barker, Michael Markl, Brigitte Stiller

**Affiliations:** 1 Department of Radiology, Northwestern University, Feinberg School of Medicine, Chicago, IL USA; 2 Department of Diagnostic and Interventional Radiology, University Hospital Würzburg, Würzburg, Germany; 3 Department of Congenital Heart Disease and Pediatric Cardiology, University Heart Center Freiburg, Freiburg, Germany; 4 Department of Anesthesiology, University Hospital Mainz, Mainz, Germany; 5 Department of Biomedical Engineering, McCormick School of Engineering, Northwestern University, Chicago, USA

**Keywords:** Marfan syndrome, Follow-up, Aorta, 4D flow cardiovascular magnetic resonance, Hemodynamics, Wall shear stress

## Abstract

**Background:**

The aim of this 4D flow cardiovascular magnetic resonance (CMR) follow-up study was to investigate longitudinal changes in aortic hemodynamics in adolescent patients with Marfan syndrome (MFS).

**Methods:**

4D flow CMR for the assessment of in-vivo 3D blood flow with full coverage of the thoracic aorta was performed twice (baseline scan t1/follow-up scan t2) in 19 adolescent MFS patients (age at t1: 12.7 ± 3.6 years, t2: 16.2 ± 4.3 years) with a mean follow-up duration of 3.5 ± 1.2 years. Ten healthy volunteers (24 ± 3.8 years) served as a control group. Data analysis included aortic blood flow visualization by color-coded 3D pathlines, and grading of flow patterns (helices/vortices) on a 3-point scale (none, moderate, severe; blinded reading, 2 observers). Regional aortic peak systolic velocities and systolic 3D wall shear stress (WSS) along the entire aortic wall were quantified. Z-Scores of the aortic root and proximal descending aorta (DAo) were assessed.

**Results:**

Regional systolic WSS was stable over the follow-up duration, except for a significant decrease in the proximal inner DAo segment (*p* = 0.02) between t1 and t2. MFS patients revealed significant lower mean systolic WSS in the proximal inner DAo compared with volunteers (0.78 ± 0.15 N/m^2^) at baseline t1 (0.60 ± 0.18 N/m^2^; *p* = 0.01) and follow-up t2 (0.55 ± 0.16 N/m^2^; *p* = 0.001). There were significant relationships (*p* < 0.01) between the segmental WSS in the proximal inner DAo, DAo Z-scores (*r* = −0.64) and helix/vortex pattern grading (*r* = −0.55) at both t1 and t2. The interobserver agreement for secondary flow patterns assessment was excellent (Cohen’s k = 0.71).

**Conclusions:**

MFS patients have lower segmental WSS in the inner proximal DAo segment which correlates with increased localized aberrant vortex/helix flow patterns and an enlarged diameter at one of the most critical sites for aortic dissection. General aortic hemodynamics are stable but these subtle localized DAo changes are already present at young age and tend to be more pronounced in the course of time.

**Electronic supplementary material:**

The online version of this article (doi:10.1186/s12968-017-0347-5) contains supplementary material, which is available to authorized users.

## Background

Marfan syndrome (MFS) is a syndromic aortopathy which promotes aortic aneurysms and dissections [1, 2]. The underlying monogenic disorder is caused by mutations in the fibrillin-1 (FBN1) gene encoding the extracellular matrix protein fibrillin-1 [3, 4]. Alterations in the FBN1 gene are responsible for reduced elasticity of the connective tissue because of abnormal interactions between fibrillin1 and cell signaling molecules, known as transforming growth factor (TGF β). This structurally altered connective tissue in the aorta makes the wall more susceptible to dilatation and dissection [5].

Since severe cardiovascular events typically occur much earlier than in non-hereditary aortopathies, it is crucial that patients diagnosed with MFS according to the Ghent nosology [6] are regularly monitored and appropriately treated to prevent and delay aortic dissection [7, 8]. According to the international guidelines, cardiovascular magnetic resonance (CMR) and echocardiography are the modalities of choice for regular monitoring of the aorta [9, 10]. However, there is a wide variety of CMR sequences including black blood images, cine SSFP images and contrast-enhanced CMR angiography, hampering standardization and reproducibility in the follow-up between different institutions [9].

In recent years, several studies using 4D flow CMR have demonstrated the usefulness of this technique for the assessment of abnormal 3D flow patterns and wall shear stress (WSS) [11–16]. Studies in patients with bicuspid aortic valve (BAV) have shown that disorganized outflow patterns due to abnormal valve opening can result in markedly altered regional WSS [11, 12, 17]. In addition, there is evidence that changes in WSS influence endothelial cell function and thus, promote vascular remodeling and vessel dilatation [18, 19].

Previous studies in adolescent MFS patients have described the presence of localized helical flow patterns in the ascending aorta (AAo) and vortical flow patterns in the proximal descending aorta (DAo) [20]. These flow patterns result in heterogeneous regional WSS distribution, with higher WSS values in the AAo compared with healthy volunteers [21]. Another study examining older, mainly adult MFS patients, also detected altered hemodynamic indices which showed significant correlations with aortic root diameter and Z-scores [22].

To our knowledge there are no 4D flow-based follow-up studies in the literature. In order to understand and detect hemodynamic changes in aortic diseases over time, however, follow-up imaging is essential. Our study aims to fill this gap by analyzing the evolution of aortic hemodynamics during longitudinal follow-up of adolescent MFS patients by 4D flow CMR. We hypothesize that there is progressive AAo dilatation associated with increasing abnormal blood flow patterns and WSS distribution in MFS patients over time.

## Methods

### Study Cohort

Nineteen pediatric/adolescent patients with confirmed MFS according to the Ghent nosology were prospectively included and underwent 4D flow CMR of the thoracic aorta at baseline (t1) and follow-up (t2). 4D flow CMR was added to the standard-of-care CMR which was clinically indicated. In addition, ten healthy young adult volunteers with a normal aortic diameter, regular valve function and without any history of cardiovascular disease served as a control group.

### MR Imaging

Patients and volunteers were scanned on a 1.5 T system (Avanto, Siemens, Germany) with a 12-channel body-phased array coil. Ten patients were examined on a 3 T MR system (Trio, Siemens, Germany) at baseline examination. 4D flow CMR (k-space segmented rf-spoiled gradient echo sequence with interleaved 3-directional velocity encoding) acquisitions were synchronized to the heart rate and breathing using prospective ECG-gating and adaptive diaphragm navigator gating. 4D flow data were acquired with full 3D coverage of the thoracic aorta and the following sequence parameters: velocity sensitivity = 150–200 cm/s, TE = 2.4–3.7 ms, TR = 4.8-6.1 ms, FOV = 210–270 mm × 275–360 mm, spatial resolution = (1.7-2.9) x (1.5–2.4) x (2.2–3.5) mm^3^, temporal resolution ~ 38–48 ms, flip angle 7° without contrast medium, 15° after contrast injection). Parallel imaging (GRAPPA) with a reduction factor of 2 was used. Scan time was about 8–12 min depending on the respiratory gating efficiency and heart rate.

Prior to 4D flow acquisitions a time-resolved contrast-enhanced MR angiography (CE-MRA) was performed in patients (0.1 mmol/kg gadoteridol; TE = 0.9–1.4 ms, TR = 2.1–3.5 ms, spatial resolution = (0.9–1.4) x (0.9–1.4) x (1.2–1.6) mm^3^, temporal resolution = 2.2–4.9 s, FA 11–25°).

### Data analysis

4D flow data were postprocessed using home-built software programmed in Matlab (The MathWorks Inc., USA) for noise reduction, velocity anti-aliasing and correction for eddy-current induced phase offset errors [23]. A 3D phase-contrast MR angiogram (PC-MRA) was generated and a peak systolic 3D segmentation of the thoracic aorta was performed (Mimics, Materialise, Belgium). 3D Visualization of aortic flow was performed by time-resolved 3D pathlines and streamlines at different time frames of the cardiac cycle (EnSight v. 9.2, CEI, Apex, NC, USA). Secondary flow patterns (helical and vortical flow) in the ascending aorta (AAo), aortic arch, and descending aorta (DAo) were analyzed by two independent experienced observers and graded in three categories: none = 0, moderate (flow rotation <360°) = 1, pronounced (flow rotation >360°) = 2. We defined a helical flow pattern as a spiral movement along the flow direction axis and a vortical flow pattern as re-circulating areas deviating from the anticipated physiological flow direction. In case of discrepancy between the readers, the value was averaged. As vortex patterns primarily occurred in the DAo and were mostly combined with a helical component, we added up the helix and vortex scores to obtain a semi quantitative parameter allowing for a better global grading of abnormal flow severity. In order to compare corresponding timing between the first and the second scan, we carefully assessed the flow patterns over the whole cardiac cycle to investigate systolic and diastolic flow alterations.

**Fig. 1:**
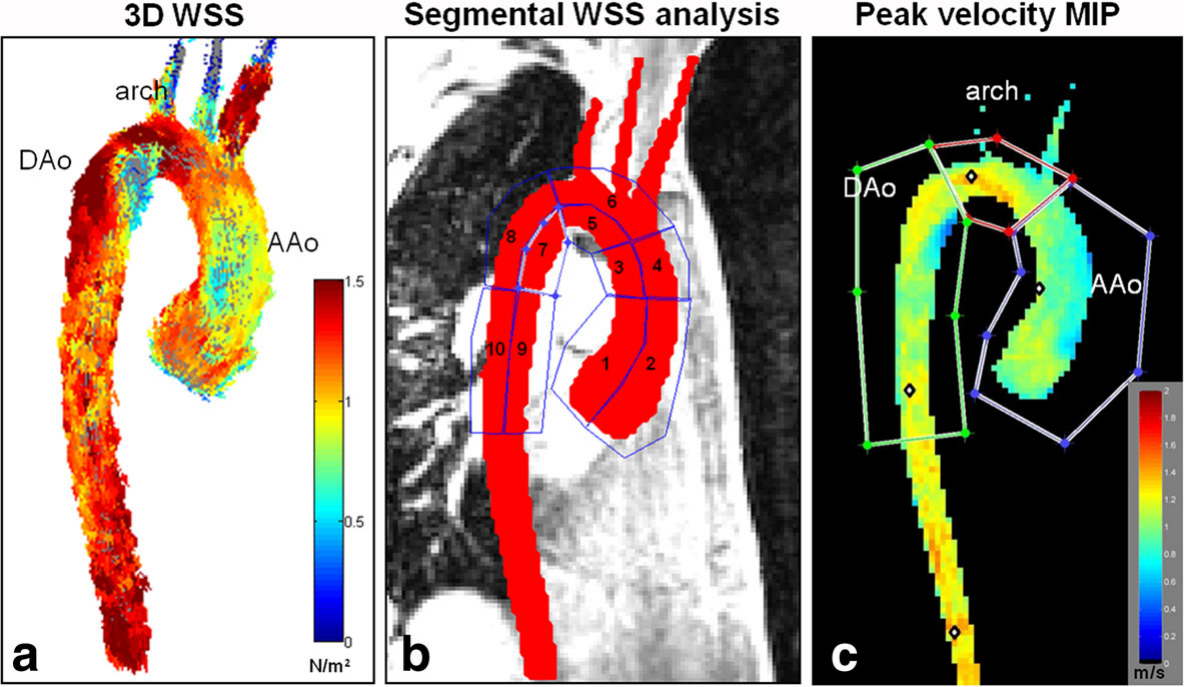
**a**) 3D WSS throughout aortic surface, **b**) 10 regions used for segmental WSS analysis along the thoracic aorta: 1 = proximal inner AAo, 2 = proximal outer AAo, 3 = distal inner AAo, 4 = distal outer AAo, 5 = inner arch, 6 = outer arch, 7 = proximal inner DAo, 8 = proximal outer DAo, 9 = distal inner DAo, 10 = distal outer DAo, **c**) Peak systolic velocity maximum intensity projection (MIP) with one erosion. AAo = ascending aorta, DAo = descending aorta

Based on the 3D segmentation, time-resolved 3D WSS along the entire aortic wall was calculated using a previously described approach (Fig. 1a) [24, 25]. The velocities within the segmented aorta were averaged for each cardiac time frame and peak systole was defined as the cardiac time frame with highest velocity. Typically, 16 to 28 cardiac phases were acquired depending on the heart rate. Time-averaged systolic values for absolute WSS (WSSsys) were defined as the average over the five cardiac time frames centered on the peak systolic time frame. Regional mean peak systolic WSS values were obtained by dividing the thoracic aorta into 10 defined segments for the AAo, arch and DAo (Fig.1b). Regional aortic peak systolic velocities were obtained from velocity maximum intensity projections (MIPs) which were mapped onto parasagittal view of the thoracic aorta for regional analysis in the AAo, arch, and DAo (Fig.1c). To assess reproducibility, regional peak systolic WSS and peak systolic velocities were analyzed in 10 patients by a second observer blinded to the first observer’s results. For segmental WSS and regional peak systolic velocity analysis, the anatomic borders were manually drawn by both observers.

### Diameter measurements

The aortic sinus diameters were measured in echocardiography (Vivid 7, General Electrics, Vingmed Ultrasound, Horton, Norway) using 2.5-5 MHz sector probes. Diameters of the mid AAo, aortic arch, proximal DAo (at the transition from the arch to the DAo) and DAo at the level of the pulmonary artery were obtained from CE-MRA data and measured on a PACS Workstation IMPAX EE (Agfa Healthcare). Multiplanar reformats of the angiogram in the cross-sectional view were used to obtain two orthogonal measurements of the vessel diameter, which were averaged.

Aortic diameters were normalized to body surface area (BSA). In addition, to account for the individual patient’s age and size, Z-scores for the aortic sinus were calculated using ultrasound-derived normative data [26]. For Z-scores of the DAo, we used a free available calculator for children specific MRA-based aortic values provided by Kaiser et al. [27].

### Statistical analysis

The assumption of normal distribution was assessed for each parameter using the Kolmogorov-Smirnov test. Quantitative values between baseline and follow-up examinations were compared using a two-sided paired *t*-test. For comparison between patients and volunteers a non-paired *t*-test was applied. In case of non-normally distributed data, a Wilcoxon rank sum test and a Mann–Whitney test were used. Interobserver variability for flow visualization was assessed using Cohen’s Kappa test (Kappa coefficient). Agreement for peak velocity and WSS analysis between observers was established by Bland-Altman analysis. To identify relationships between hemodynamic parameters (WSS, velocity, flow patterns) and Z-scores, Pearson correlation coefficient was calculated. All tests used a significance level of *p* < 0.05. Statistical analysis was performed using SPSS Statistics 17 (IBM Corporation).

## Results

### Study cohort

4D flow CMR examinations were successfully performed in all patients and volunteers. Mean age of the 19 MFS patients (9 male, 10 female) at baseline t1 was 12.7 ± 3.6 years, range 2–17 years; at follow-up t2 16.2 ± 4.3 years, range 4–22 years. Mean follow-up duration was 3.5 ± 1.2 years, range 1.9–6.1 years. Ten patients were treated with beta blockers or losartane. The youngest patient, aged 2 at baseline and 4 years at follow-up, was imaged in general anesthesia performed under the guidance of a pediatric anesthesiologist. Patient demographics are summarized in Table 1. The ten healthy young adult volunteers (4 male, 6 female) had a mean age of 24.2 ± 3.8 years, range 19–29 years.

**Table 1:** Demographics of the MFS study population

	Age [years]	Sex	Weight [kg]	Height [cm]	BSA [m^2^]	Heart rate [bpm]
t1	12.7 ± 3.6 median = 13	10 female	54.0 ± 17.2	172.0 ± 21.9	1.59 ± 0.35	70.3 ± 14.8
t2	16.2 ± 4.3 median = 17	10 female	64.4 ± 16.5	181.1 ± 17.2	1.79 ± 0.32	68.4 ± 14.3

*BSA* body surface area, *bpm* beats per minute

### Aortic diameters

Using a Z-score > 2 to define dilation, 10/19 patients had aortic root dilatation at both examinations (average t1: Z = 2.11 ± 1.37, t2: Z = 2.05 ± 1.18). The Z-score of the proximal DAo increased significantly within the follow-up period from 3.15 ± 1.66 to 3.71 ± 1.9 (*p* < 0.001), although the BSA-normalized diameters of the proximal DAo decreased at follow-up. 13 patients had a segmental DAo dilatation at t1, and 14 at t2. Normalized AAo and arch diameters decreased at t2 (Table 2).

**Table 2:** Aortic diameters in MFS patients at baseline t1 and follow-up t2

Diameters	MFS t1	MFS t2	*p*-value
AAo [mm/m^2^]	15.7 ± 4.3	14.6 ± 2.8	0.072
arch [mm/m^2^]	12.7 ± 2.5	12.0 ± 1.9	**0.030**
DAo [mm/m^2^]	12.3 ± 3.0	13.0 ± 2.0	0.255
Aortic sinus [mm/m^2^]	20.7 ± 2.6	19.6 ± 2.6	**0.005**
Aortic sinus Z-Score	2.11 ± 1.37	2.05 ± 1.18	0.72
Proximal DAo [mm/m^2^]	14.4 ± 2.9	13.8 ± 2.3	**0.02**
Proximal DAo Z-Score	3.15 ± 1.66	3.71 ± 1.90	**<0.001**

*AAo* ascending aorta, *DAo* descending aorta, *MFS* Marfan syndrome

Statistically significant differences are highlighted in bold

### Regional WSS distribution and peak velocities

**Fig. 2:**
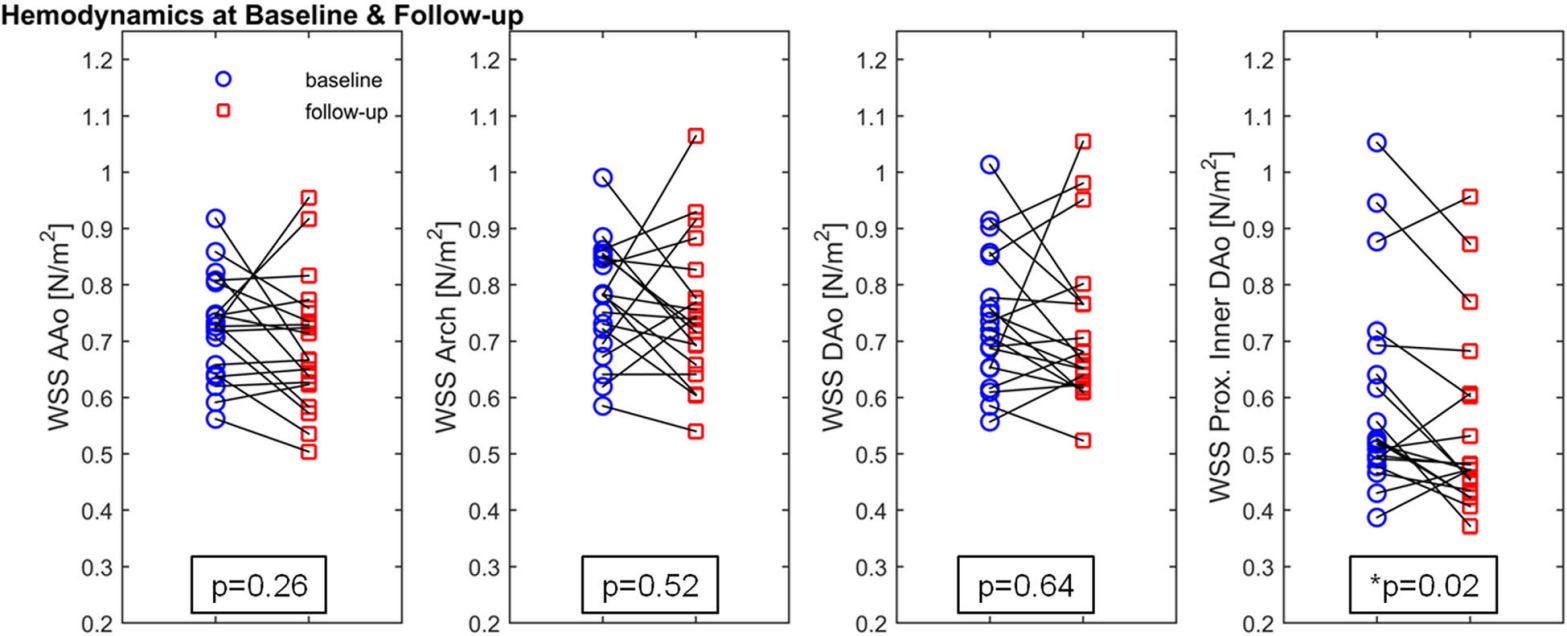
Segmental mean systolic WSS at baseline and follow-up in MFS patients for the ascending aorta, arch, descending aorta, and the proximal inner segment of the descending aorta, AAo = ascending aorta, DAo = descending aorta, * indicates significant difference, *p*<0.05

**Fig. 3:**
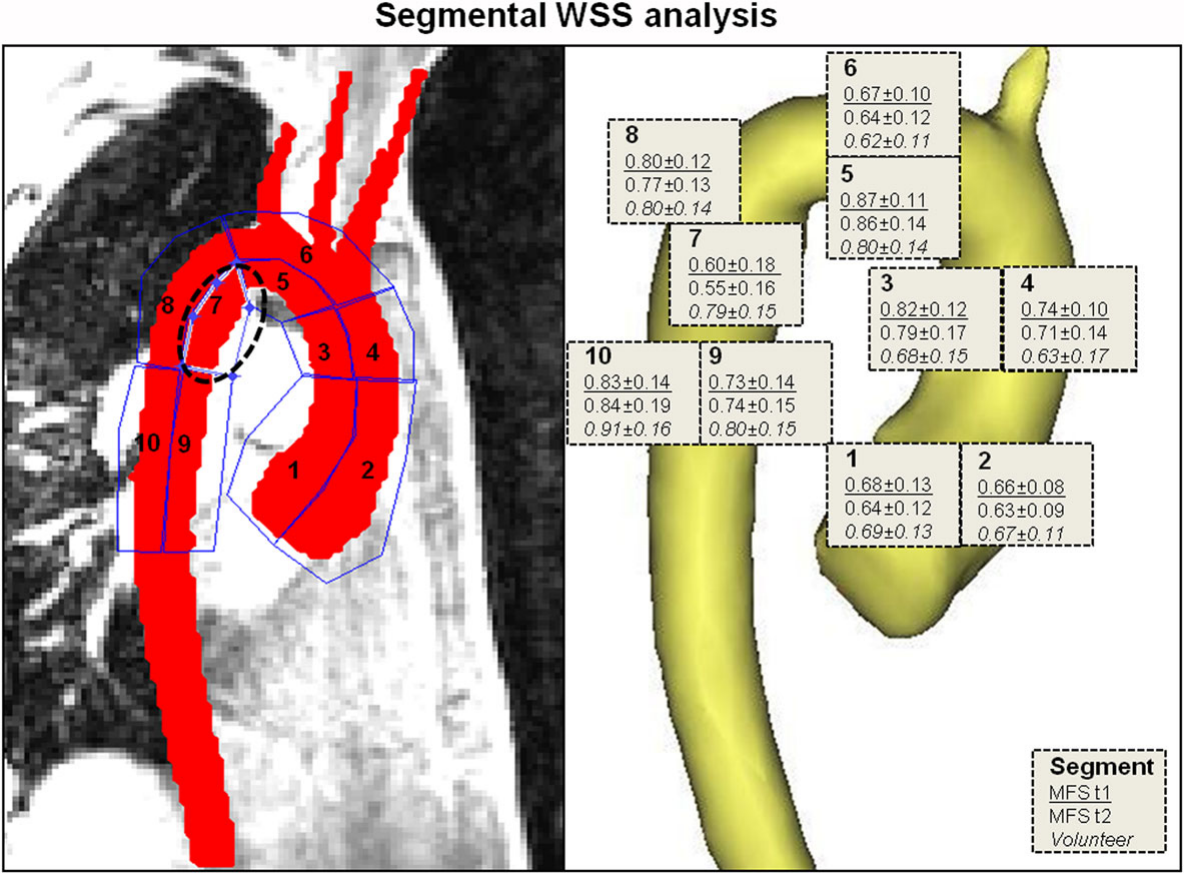
Segmental WSS in the 10 segments along the thoracic aorta mapped on the anatomic thoracic background (left): 1 = proximal inner AAo, 2 = proximal outer AAo, 3 = distal inner AAo, 4 = distal outer AAo, 5 = inner arch, 6 = outer arch, 7 = proximal inner DAo, 8 = proximal outer DAo, 9 = distal inner DAo, 10 = distal outer DAo. WSS values of the 10 analyzed segments at baseline t1 and follow-up t2 for MFS patients and volunteers (right)

As shown in Table 3, Figs. 2 and 3, segmental mean systolic WSS in MFS patients was heterogeneous between patients, and did not change significantly between baseline and follow-up, except for a significant decrease in the proximal inner DAo segment (t1: 0.60 ± 0.18 N/m^2^, t2: 0.55 ± 0.16 N/m^2^; *p* = 0.02). This aortic segment was also the only one which revealed significant lower mean systolic WSS compared with volunteers (0.78 ± 0.15 N/m^2^) at both scans (t1: *p* = 0.01, t2: *p* = 0.001). In addition, mean systolic WSS in MFS patients was significantly different between the proximal inner and outer AAo and DAo segment at both scans (*p* < 0.001), which was not observed in volunteers. After subdividing the AAo and DAo into their proximal and distal segments, significant differences were found in the patient group in terms of lower WSS values in the proximal compared with the distal AAo and DAo (AAo: *p* < 0.001, DAo: *p* = 0.001), whereas no significant differences were found in volunteers.

**Table 3:** Aortic hemodynamics in patients with MFS and volunteers

Hemodynamics	MFS t1	MFS t2	volunteers	*p*-value
WSS AAo [N/m^2^]	0.72 ± 0.09	0.69 ± 0.12	0.67 ± 0.13	* 0.26; ^ 0.23; # 0.6
WSS prox. inner AAo [N/m^2^]	0.68 ± 0.13	0.64 ± 0.12	0.69 ± 0.13	* 0.15; ^ 0.85; # 0.26
WSS prox. outer AAo [N/m^2^]	0.66 ± 0.08	0.63 ± 0.09	0.67 ± 0.11	* 0.22; ^ 0.85; # 0.39
WSS dist. inner AAo [N/m^2^]	0.82 ± 0.12	0.79 ± 0.17	0.68 ± 0.15	* 0.44; ^ **0.02**; # 0.09
WSS dist. outer AAo [N/m^2^]	0.74 ± 0.10	0.71 ± 0.14	0.63 ± 0.17	* 0.48; ^ 0.09; # 0.20
WSS arch [N/m^2^]	0.77 ± 0.10	0.75 ± 0.13	0.71 ± 0.13	* 0.52; ^ 0.59, # 0.41
WSS inner arch [N/m^2^]	0.87 ± 0.11	0.86 ± 0.14	0.80 ± 0.14	* 0.72; ^ 0.17; # 0.27
WSS outer arch [N/m^2^]	0.67 ± 0.10	0.64 ± 0.12	0.62 ± 0.11	* 0.37; ^ 0.26; # 0.64
WSS DAo [N/m^2^]	0.74 ± 0.12	0.72 ± 0.14	0.82 ± 0.14	*0.64; ^ 0.14; # 0.09
WSS prox. inner DAo [N/m^2^]	0.60 ± 0.18	0.55 ± 0.16	0.78 ± 0.15	* 0.02; ^ 0.01; # 0.001
WSS prox. outer DAo [N/m^2^]	0.80 ± 0.12	0.77 ± 0.13	0.80 ± 0.14	* 0.49; ^ 0.91; # 0.55
WSS dist. inner DAo [N/m^2^]	0.73 ± 0.14	0.74 ± 0.15	0.80 ± 0.15	* 0.72; ^ 0.2; # 0.29
WSS dist. outer DAo [N/m^2^]	0.83 ± 0.14	0.84 ± 0.19	0.91 ± 0.16	* 0.87; ^ 0.2; # 0.29
Max. velocity AAo [m/s]	1.50 ± 0.19	1.38 ± 0.19	1.45 ± 0.16	* 0.02; ^ 0.07; # 0.36
Max. velocity arch [m/s]	1.41 ± 0.17	1.32 ± 0.19	1.16 ± 0.15	* 0.06; **^ <0.001; # 0.02**
Max. velocity DAo [m/s]	1.57±0.26	1.48±0.16	1.39±0.23	* 0.21; ^ 0.07; # 0.32

*WSS* wall shear stress, *AAo* ascending aorta, *DAo* descending aorta, *prox*. proximal, *dist*. distal, *max*. maximum, *MFS* Marfan syndrome. Statistically significant differences are highlighted in bold

*p-value between MFS t1 and t2; ^ *p*-value between MFS t1 and volunteers, # *p*-value between MFS t2 and volunteers

Peak systolic flow velocity in the AAo decreased significantly between t1 and t2 (t1: 1.50 ± 0.19 m/s; t2: 1.38 ± 0.19 m/s; *p* = 0.02), but was not different compared with volunteers (1.45 ± 0.16 m/s). Maximum arch velocity was significantly higher in MFS patients at t1 (*p* < 0.001) and t2 (*p* = 0.02) in comparison with volunteers, whereas DAo velocity was similar. Details are provided in Table 3.

### Flow visualization

**Fig. 4:**
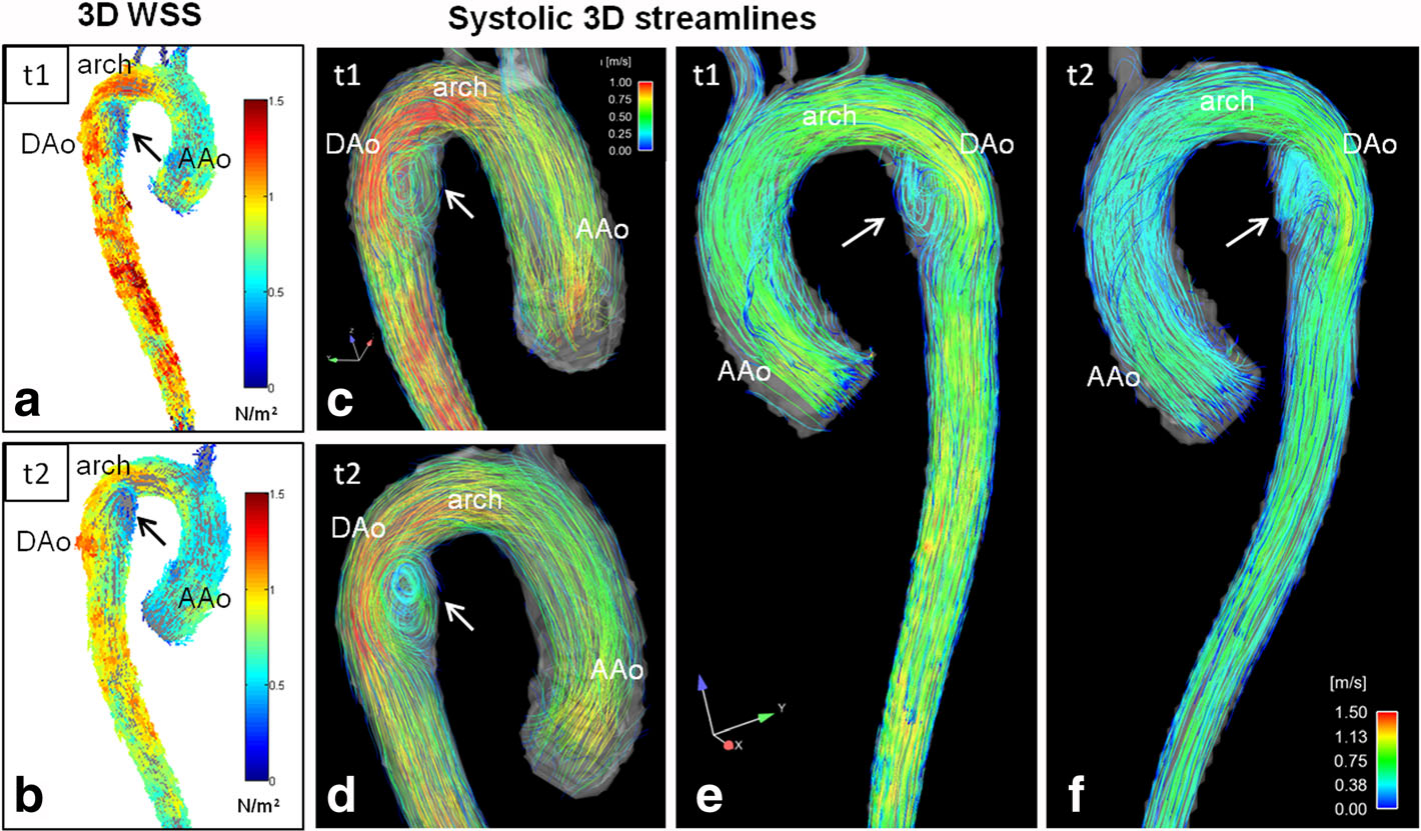
Hemodynamics in a Marfan patient at baseline t1 and at follow-up t2 after 3 years. a) and b): WSS distribution along the aortic surface at t1 (**a**) and t2 (**b**) depicts the segmental low WSS at the inner curvature of the proximal descending aorta (black arrows). C)-f) reveal aortic flow at late systole/early diastole visualized by streamlines, **c**) and **d**) posterior view, e) and f) anterior view. Along with low WSS, marked combined helix/vortex flow patterns (white arrows) in the dilated proximal descending aorta were scored as grade 2.5 at t1 (c and e) and 3 at t2 (d and f). AAo = ascending aorta, DAo = descending aorta

**Fig. 5:**
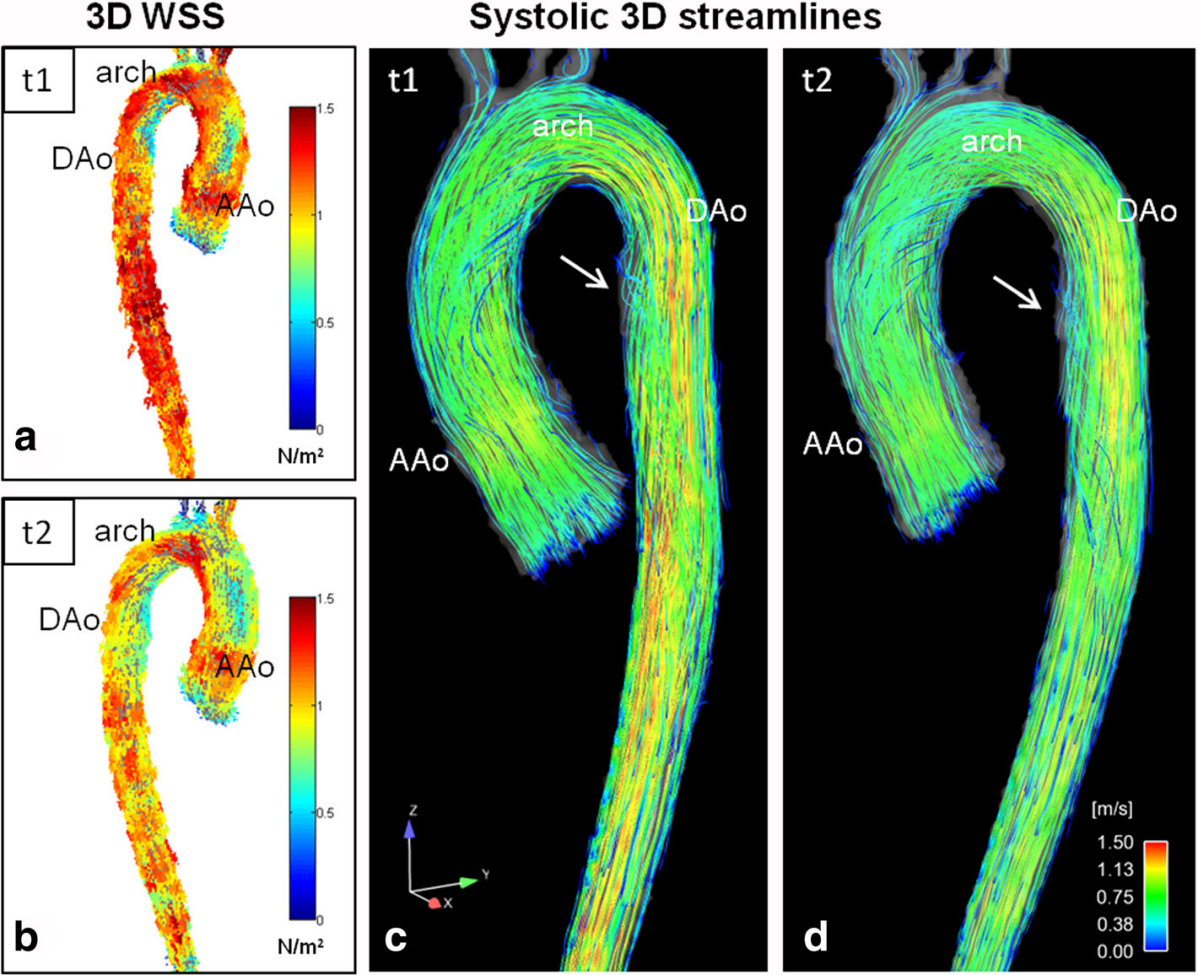
Hemodynamics in a Marfan patient at baseline t1 and at follow-up t2 after 3 years. a) and b): WSS along the aortic surface at t1 (a) and t2 (b) depicts a normal WSS distribution which was consistent with volunteers. C) and d): flow visualization by streamlines during late systole/early diastole. There are only slight helices (white arrows) in the normal sized DAo which were assessed as grade 1 both at t1 and t2. AAo = ascending aorta, DAo = descending aorta



The interobserver agreement for secondary flow patterns assessment was excellent (Cohen’s k = 0.71). Vortical flow patterns were only observed in MFS patients in the proximal DAo and were more frequent at t2 (t1: 9/19, t2: 12/19 patients). Aberrant flow patterns (helices + vortices) at the inner curvature at the transition from the arch to the DAo showed a significant increase at follow-up (grade at t1: 1.61 ± 1.04, t2: 2.0 ± 0.85, *p* = 0.03). Examples of DAo vortical flow patterns at baseline and follow-up are shown in Fig. 4 and movie 1 compared to a patient without evident flow alterations (Fig. 5). An additional movie file shows this in more detail [see Additional file 1]. Localized helical flow patterns in the AAo decreased significantly between t1 and t2 (*p* = 0.04). None of the volunteers had localized helical or vortical flow patterns.

**Additional file 1:** Hemodynamics in a Marfan patient at baseline t1 (same patient as in Fig. 4). Blood flow visualization by color-coded pathlines according to velocity reveals helical and vortical flow patterns in the proximal descending aorta. (MPG 2236 kb)


### Hemodynamic associations

As expected, AAo systolic WSS and peak velocities were significantly associated in both MFS patients and volunteers (*r* = 0.62 at t1, *r* = 0.78 at t2, r = 0.80 in volunteers, *p* < 0.01). We also found a high correlation between the maximum DAo velocities and the WSS in the proximal inner DAo segment in volunteers (*r* = 0.87, *p* = 0.01), which was less pronounced for patients at t2 (*r* = 0.45, *p* = 0.05) and even not present at t1 (*r* = 0.10, *p* = 0.7).

The segmental WSS in the proximal inner DAo had a significant negative association with the helix/vortex grading at both t1 and t2 (t1: *r* = −0.51, *p* = 0.025; t2: *r* = −0.56, *p* = 0.014). Moreover, we detected significant relationships between the segmental WSS in this region and the DAo Z-scores for baseline and follow-up (t1: r = −0.60, *p* = 0.007; t2: *r* = −0.66, *p* = 0.002). The relationship between the segmental WSS at t1 and the Z-score at t2 was even closer (*r* = −0.74, *p* < 0.001). In addition, there were high correlations between DAo helix/vortex and DAo Z-score (t1: *r* = 0.59, *p* = 0.008; t2: *r* = 0.79, *p* < 0.001) (Fig. 4 in comparison to Fig. 5).

**Fig. 6:**
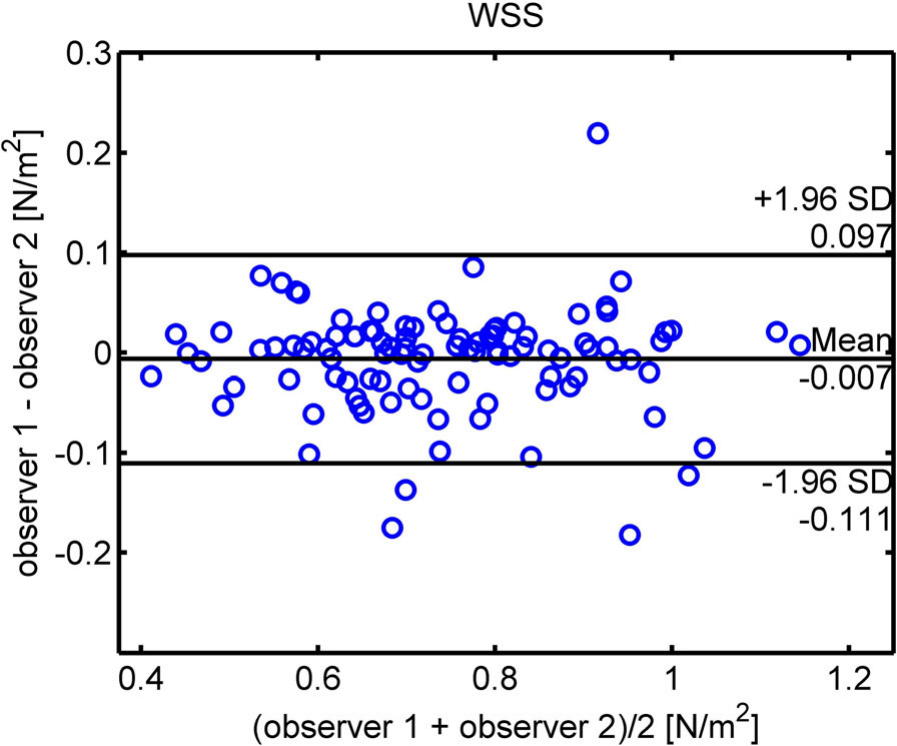
Bland-Altman diagram of the WSS interobserver study

Bland-Altman analysis for segmental WSS calculation in all 10 segments revealed good interobserver correlation with a mean bias of −0.007 N/m^2^, or 1% of average value of 0.74 N/m^2^. Upper and lower limits of agreement were 0.097 N/m^2^ (13%) and −0.111 N/m^2^ (15%), respectively (Fig. 6). Interobserver reproducibility for peak velocity in AAo, arch and DAo was excellent with less than 1% mean difference of average values (1.51 m/s) and 3% upper and lower limits of agreement.

## Discussion

The results of our 4D flow CMR follow-up study in MFS patients demonstrated generally stable hemodynamic findings in the thoracic aorta in a 3-year follow-up. However, we found a significant decrease in regional mean systolic WSS in the inner segment of the proximal DAo at baseline and follow-up examinations, which were both significantly different than normal values obtained from healthy volunteers. Based on a segment-wise analysis of the complete thoracic aorta, this region was the only one showing significant WSS changes in the follow-up. Interestingly, low WSS values were associated with abnormal localized flow patterns and enlarged diameters of the proximal DAo expressed by pathological Z-scores. These findings were more pronounced in the follow-up, whereas results in the aortic root and AAo in terms of diameter, WSS and flow patterns did not show significant changes between baseline and follow-up. This is an important and intriguing finding, as the proximal DAo is a known initiation region for type B aortic dissections.

Previous 4D flow CMR studies on MFS patients had mainly described WSS alterations in the AAo, which were partly contradictory probably due to different age ranges [21, 22]. While the study on adolescent MFS patients with a mean age of 18 years revealed an inhomogeneous WSS distribution in the AAo with localized increased values in the inner curvature of the proximal AAo [21], a more recent study (patients’ mean age 26 years) reported lower WSS in the AAo and arch in comparison with age-matched controls [22]. In contrast to our results, Wang et al. did not find abnormal flow patterns or decreased segmental WSS in the proximal DAo in their MFS cohort [22]. In addition, they described a relatively symmetric WSS distribution compared with values of normal subjects, which is also discordant with our results. We found significant differences not only in the proximal DAo but also in WSS values between proximal and distal AAo segments, as well as between outer and inner distal AAo segments expressing inhomogeneous WSS distribution throughout the AAo.

However, both aforementioned studies used a plane-wise approach to calculate regional WSS, which doesn’t take advantage of the three-dimensional coverage of the acquired 4D flow data and can therefore miss regional information located between the 2D planes [28]. In addition, this technique is more prone to individual and observer dependent errors due to manual vessel contour segmentation. Recently developed methods to compute 3D WSS in the thoracic aorta showed very promising results in characterizing abnormal WSS in patients with bicuspid aortic valve (BAV) compared to healthy volunteers, and low interobserver variability in a reproducibility study in healthy volunteers [24, 25, 29, 30]. Although we know that there is individual variability in segmental WSS distribution, it is remarkable that WSS in the proximal inner DAo segment was below 0.5 N/m^2^ in 12/17 patients at the second scan (mean 0.55 N/m^2^), whereas segmental WSS was above 0.5 N/m^2^ (mean 0.78 N/m^2^) in all volunteers.

3D WSS could be reliably assessed even in the youngest patients of our cohort, aged 2 and 4 years having smaller aortic diameters than the majority which consisted of teenagers.

One previous 4D flow study on MFS patients found an increased number of helices and vortices in the proximal DAo besides frequent localized AAo helices in the presence of mildly enlarged aortic root diameters [20]. Since flow patterns may be very complex, it can sometimes be challenging to differentiate vortical from helical flow patterns. In order to achieve semi objective grading, we decided to sum up either flow pattern (vortical and helical) which we observed in the DAo. Based on our experience, mild helical patterns can be detected in the normal population’s DAo, as well. In our study, 17/19 patients had at least a grade 1 helix/vortex at t1 and all patients at t2, with an obvious increase in the combined helix/vortex severity at follow-up. At the same time, the mean DAo Z-score increased despite the length growth respectively a change in BSA during puberty, and was above 2 in those patients with at least a grade 2 for helix/vortex in the proximal DAo. The re-circulating blood seems to have a negative impact on the vessel wall at the inner curvature of the proximal DAo, resulting in localized decreased WSS and concomitant vessel enlargement. According to our results we hypothesize that a flow grading of 2 or greater represents pathological flow.

A number of studies have investigated aortopathy, which were mainly focused on hemodynamic alterations in the AAo when concerning altered flow patterns and WSS parameters in the presence of aortic dilatation [11, 12, 15, 31–33]. We know from these studies that there is a complex interaction between these factors influencing one another, and making it rather challenging to predict which factor precedes the others in disease progression. This is also an ongoing discussion regarding the vasculature in other body regions like intracranial or abdominal aorta aneurysms, in which either high or low WSS is reported to trigger unfavorable pathways leading to vessel wall remodeling [18, 34].

Only few studies have focused their investigation on the thoracic DAo, which is known to be the second most critical region for aortic dissection in MFS patients [35, 36]. Mimoun et al. reported that the rate of type B dissections was independent from aortic root dilatation but was associated with larger DAo diameters [35]. This was confirmed by a larger study which highlighted the importance of the proximal DAo diameter with a cutoff value of 27 mm as an independent factor for type B dissection [36]. According to this, 8/19 patients would have reached this cutoff value in our study at t2. Previous valve sparing root replacement was also associated with a higher risk of developing aneurysms and dissections in the DAo [7, 36]. A study in a small cohort of 12 MFS patients after root replacement described slight DAo helices in half of the patients, and abnormal flow and decreased WSS in one patient who developed a type B dissection [37].

These findings confirm our observations on MFS patients and support the hypothesis that hemodynamic alterations in the proximal DAo represent a risk factor for type B dissections. It is very interesting that the patients of our cohort displayed pathological findings already at young age, years before dissections’ occurrence. We had expected to observe more abnormalities in the AAo, however, the main findings in our cohort were located in the proximal DAo. Although the majority had enlarged aortic root diameters, the DAo Z-scores were more notable in the follow-up period. It is important to normalize the values to the BSA or to use Z-scores, particularly in children and adolescents, for a more objective comparison of the measurements in the follow-up. We used two different approaches to calculate Z-scores because of the different employed imaging modalities, aortic root diameters being measured in echocardiography whereas the DAo diameters were measured in MR images. The contradictory findings with regard to BSA values and Z-scores could either be explained by the used regression method by Kaiser et al. [27] or the nonconformity between echocardiography and CMR proposed reference values. The rapid length growth of our patient cohort’s majority during puberty could also lead to this discrepancy, since MFS patients are frequently taller than the general population and thus, than their peers. A fact that results in a decrease in normalized BSA values despite increased aortic diameters at follow-up, but increased Z-scores, which sum up the difference between the observed and the expected measurement divided by the standard deviation.

The overall importance of the DAo is still under-recognized in the MFS population, but it may increase in the future owing to the progress to prevent type A dissection by more aggressive prophylactic surgery in recent years [5]. Several studies have shown the protective character of angiotensin-II-inhibitors to avoid rapid progressive aortic dilatation and dissection [36, 38]. However, it remains unclear why there is a discrepancy in evolution between the aortic root and DAo diameter in our patient cohort. One important aspect in this context is the fact that multiple FBN1-mutations have been identified so far, which result in various phenotypes and thus, in unpredictable genotype-phenotype correlation [2]. The MFS population consists of a rather heterogeneous group of individuals who seem to have individual risks for dissection. This is also evident in a pair of siblings in our MFS cohort shown in Figs. 4 and 5. There seem to be patients with the typical AAo pattern and patients with combined AAo and DAo abnormalities; however, in our cohort, most of the patients had a predominant DAo pathology.

Our findings suggest that elevated helical and vortical flow patterns above grade 2 and concomitant low segmental WSS below 0.5 N/m^2^ may mark a threshold that could place a patient at higher risk of progressive disease and early dissection.

Limitations of our study include the small patient cohort and the age difference between patients and controls. We know that peak systolic velocity and WSS decrease with increasing age [39]. Therefore, we chose the youngest available volunteer group (19–29 years) as a reference cohort to account for sensitivity to age. Age-matched healthy volunteers were not available due to IRB restrictions. However, since WSS is derived from velocity and that we did not find significant differences in maximum velocity between patients and controls, our WSS results might indeed reflect disease-related alterations, independent of the age difference between the 2 populations. The excellent interobserver-agreement also supports the robustness of the 3D WSS calculation. Despite manually drawn regions of interest for WSS and peak systolic velocity assessment, only minor inter-operator disagreements concerning the anatomic borders occurred. Differences in field strength (1.5 vs 3 T) did not have an impact on image quality and further flow assessment.

Our results are influenced by the antihypertensive medication which results in changes in cardiac output. Most of the patients had a considerable length growth during puberty between the baseline and follow-up examinations; this is another factor which has an impact on vascular size and the cardiovascular system. Therefore, it would be interesting to re-examine the full-grown patients in the critical period between the age of 20 and 30 years prior to the occurrence of dissections.

## Conclusions

4D flow CMR in adolescent MFS patients showed overall stable aortic hemodynamics in a 3-year follow-up. However, we detected conspicuous changes in the proximal inner segment of the DAo in terms of diameter, flow and WSS. These abnormalities tend to become more manifest in the follow-up and are located at a critical site for type B aortic dissections.

## Abbreviations

4D flow CMR: Three-dimensional cine phase contrast magnetic resonance imaging with 3-directional velocity encoding
AAo: Ascending aorta
BSA: Body surface area
DAo: Descending aorta
MFS: Marfan syndrome
MIP: Maximum intensity projections
PC-MRA: Phase contrast magnetic resonance angiogram
WSS: Wall shear stress

## References

[1] Isselbacher EM, Lino Cardenas CL, Lindsay ME (2016). Hereditary Influence in Thoracic Aortic Aneurysm and Dissection. Circulation.

[2] Caglayan AO, Dundar M (2009). Inherited diseases and syndromes leading to aortic aneurysms and dissections. Eur J Cardiothorac Surg.

[3] Judge DP, Dietz HC (2005). Marfan’s syndrome. Lancet.

[4] Sakai LY, Keene DR, Renard M, De Backer J (2016). FBN1: The disease-causing gene for Marfan syndrome and other genetic disorders. Gene.

[5] Dormand H, Mohiaddin RH (2013). Cardiovascular magnetic resonance in Marfan syndrome. J Cardiovasc Magn Reson.

[6] Loeys BL, Dietz HC, Braverman AC, Callewaert BL, De Backer J, Devereux RB (2010). The revised Ghent nosology for the Marfan syndrome. J Med Genet.

[7] Engelfriet PM, Boersma E, Tijssen JG, Bouma BJ, Mulder BJ (2006). Beyond the root: dilatation of the distal aorta in Marfan’s syndrome. Heart.

[8] Ammash NM, Sundt TM, Connolly HM (2008). Marfan syndrome-diagnosis and management. Curr Probl Cardiol.

[9] Baumgartner H, Bonhoeffer P, De Groot NM, de Haan F, Deanfield JE, Galie N (2010). ESC Guidelines for the management of grown-up congenital heart disease (new version 2010). Eur Heart J.

[10] Hiratzka LF, Bakris GL, Beckman JA, Bersin RM, Carr VF, Casey DE (2010). 2010 ACCF/AHA/AATS/ACR/ASA/SCA/SCAI/SIR/STS/SVM guidelines for the diagnosis and management of patients with Thoracic Aortic Disease: a report of the American College of Cardiology Foundation/American Heart Association Task Force on Practice Guidelines, American Association for Thoracic Surgery, American College of Radiology, American Stroke Association, Society of Cardiovascular Anesthesiologists, Society for Cardiovascular Angiography and Interventions, Society of Interventional Radiology, Society of Thoracic Surgeons, and Society for Vascular Medicine. Circulation.

[11] Barker AJ, Markl M, Burk J, Lorenz R, Bock J, Bauer S (2012). Bicuspid aortic valve is associated with altered wall shear stress in the ascending aorta. Circ Cardiovasc imaging.

[12] Allen BD, van Ooij P, Barker AJ, Carr M, Gabbour M, Schnell S (2015). Thoracic aorta 3D hemodynamics in pediatric and young adult patients with bicuspid aortic valve. J Magn Reson Imaging.

[13] Hope MD, Hope TA, Meadows AK, Ordovas KG, Urbania TH, Alley MT (2010). Bicuspid aortic valve: four-dimensional MR evaluation of ascending aortic systolic flow patterns. Radiology.

[14] Francois CJ, Markl M, Schiebler ML, Niespodzany E, Landgraf BR, Schlensak C (2013). Four-dimensional, flow-sensitive magnetic resonance imaging of blood flow patterns in thoracic aortic dissections. J Thorac Cardiovasc Surg.

[15] Bieging ET, Frydrychowicz A, Wentland A, Landgraf BR, Johnson KM, Wieben O (2011). In vivo three-dimensional MR wall shear stress estimation in ascending aortic dilatation. J Magn Reson Imaging.

[16] Frydrychowicz A, Markl M, Hirtler D, Harloff A, Schlensak C, Geiger J (2011). Aortic hemodynamics in patients with and without repair of aortic coarctation: in vivo analysis by 4D flow-sensitive magnetic resonance imaging. Investig Radiol.

[17] Burris NS, Hope MD (2015). 4D flow MRI applications for aortic disease. Magn Reson Imaging Clin N Am.

[18] Meng H, Tutino VM, Xiang J, Siddiqui A (2014). High WSS or low WSS? Complex interactions of hemodynamics with intracranial aneurysm initiation, growth, and rupture: toward a unifying hypothesis. AJNR Am J Neuroradiol.

[19] Reneman RS, Arts T, Hoeks AP (2006). Wall shear stress--an important determinant of endothelial cell function and structure--in the arterial system in vivo. Discrepancies with theory. J Vasc Res.

[20] Geiger J, Markl M, Herzer L, Hirtler D, Loeffelbein F, Stiller B (2012). Aortic flow patterns in patients with Marfan syndrome assessed by flow-sensitive four-dimensional MRI. J Magn Reson Imaging.

[21] Geiger J, Arnold R, Herzer L, Hirtler D, Stankovic Z, Russe M (2013). Aortic wall shear stress in Marfan syndrome. Magn Reson Med.

[22] Wang HH, Chiu HH, Tseng WI and Peng HH. Does altered aortic flow in marfan syndrome relate to aortic root dilatation? J Magn Reson Imaging. 2016;44(2):500–8. 10.1002/jmri.25174 PMC5132207 26854646

[23] Bock JKB, Hennig J, Markl M (2007). Optimized pre-processing of time-resolved 2D and 3D phase contrast MRI data.

[24] Potters WV, van Ooij P, Marquering H, vanBavel E, Nederveen AJ (2015). Volumetric arterial wall shear stress calculation based on cine phase contrast MRI. J Magn Reson Imaging.

[25] van Ooij P, Potters WV, Nederveen AJ, Allen BD, Collins J, Carr J (2015). A methodology to detect abnormal relative wall shear stress on the full surface of the thoracic aorta using four-dimensional flow MRI. Magn Reson Med.

[26] Sluysmans T, Colan SD (2005). Theoretical and empirical derivation of cardiovascular allometric relationships in children. J Appl Physiol.

[27] Kaiser T, Kellenberger CJ, Albisetti M, Bergstrasser E, Valsangiacomo Buechel ER (2008). Normal values for aortic diameters in children and adolescents--assessment in vivo by contrast-enhanced CMR-angiography. J Cardiovasc Magn Reson.

[28] Stalder AF, Russe MF, Frydrychowicz A, Bock J, Hennig J, Markl M (2008). Quantitative 2D and 3D phase contrast MRI: optimized analysis of blood flow and vessel wall parameters. Magn Reson Med.

[29] van Ooij P, Potters WV, Collins J, Carr M, Carr J, Malaisrie SC (2015). Characterization of abnormal wall shear stress using 4D flow MRI in human bicuspid aortopathy. Ann Biomed Eng.

[30] van Ooij P, Powell AL, Potters WV, Carr JC, Markl M, Barker AJ (2016). Reproducibility and interobserver variability of systolic blood flow velocity and 3D wall shear stress derived from 4D flow MRI in the healthy aorta. J Magn Reson Imaging.

[31] Hope MD, Hope TA, Crook SE, Ordovas KG, Urbania TH, Alley MT (2011). 4D flow CMR in assessment of valve-related ascending aortic disease. J Am Coll Cardiol Img.

[32] Burk J, Blanke P, Stankovic Z, Barker A, Russe M, Geiger J (2012). Evaluation of 3D blood flow patterns and wall shear stress in the normal and dilated thoracic aorta using flow-sensitive 4D CMR. J Cardiovasc Magn Reson.

[33] Bissell MM, Hess AT, Biasiolli L, Glaze SJ, Loudon M, Pitcher A (2013). Aortic dilation in bicuspid aortic valve disease: flow pattern is a major contributor and differs with valve fusion type. Circ Cardiovasc Imaging.

[34] Tanweer O, Wilson TA, Metaxa E, Riina HA, Meng H (2014). A comparative review of the hemodynamics and pathogenesis of cerebral and abdominal aortic aneurysms: lessons to learn from each other. J Cerebrovasc Endovasc Neurosurg.

[35] Mimoun L, Detaint D, Hamroun D, Arnoult F, Delorme G, Gautier M (2011). Dissection in Marfan syndrome: the importance of the descending aorta. Eur Heart J.

[36] den Hartog AW, Franken R, Zwinderman AH, Timmermans J, Scholte AJ, van den Berg MP (2015). The risk for type B aortic dissection in Marfan syndrome. J Am Coll Cardiol.

[37] Hope TA, Kvitting JP, Hope MD, Miller DC, Markl M, Herfkens RJ (2013). Evaluation of Marfan patients status post valve-sparing aortic root replacement with 4D flow. Magn Reson Imaging.

[38] Lacro RV, Dietz HC, Sleeper LA, Yetman AT, Bradley TJ, Colan SD (2014). Atenolol versus losartan in children and young adults with Marfan’s syndrome. N Engl J Med.

[39] van Ooij P, Garcia J, Potters WV, Malaisrie SC, Collins JD, Carr JC (2016). Age-related changes in aortic 3D blood flow velocities and wall shear stress: Implications for the identification of altered hemodynamics in patients with aortic valve disease. J Magn Reson Imaging.

